# Effects of tubeimoside-1 on the proliferation and apoptosis of BGC823 gastric cancer cells *in vitro*

**DOI:** 10.3892/ol.2013.1117

**Published:** 2013-01-08

**Authors:** YI ZHANG, XIAO-MAN XU, MENG ZHANG, DAN QU, HUI-YAN NIU, XUE BAI, LIANG KAN, PING HE

**Affiliations:** 1 Departments of Geriatrics, Shengjing Hospital of China Medical University, Shenyang, Liaoning 110004, P.R. China; 2 Respiratory Medicine, Shengjing Hospital of China Medical University, Shenyang, Liaoning 110004, P.R. China

**Keywords:** tubeimoside-1, BGC823 gastric cancer cells, natural compound, apoptosis

## Abstract

Natural products isolated from Chinese medicinal herbs are useful sources of new drugs for cancer therapy. Tubeimoside-1 (TBMS1) is a natural compound isolated from the Chinese medicinal herb *Bolbostemma paniculatum* (Maxim.) Franquet (*Cucurbitaceae*). Studies have shown that TBMS1 has anticancer effects in various human cancer cell lines. However, the effect of TBMS1 on human gastric cancer cells is unknown. In the present study, it was observed that TBMS1 inhibited BGC823 gastric cancer cell proliferation in a concentration- and time-dependent manner. Fluorescent microscopy and flow cytometric analysis showed that TBMS1 induced BGC823 cell apoptosis in a concentration-dependent manner. Western blot analysis also showed that TBMS1 induced apoptosis by regulation of the Bcl-2 gene family in BGC823 cells. These findings indicate that TBMS1 may be developed as a possible therapeutic agent for the management of gastric cancer.

## Introduction

Gastric cancer is the fourth most common type of cancer and the second most common cause of cancer-related mortality worldwide (1,2). At present, the management of gastric cancer includes surgery, radiotherapy, conventional chemotherapy, molecular targeted therapy and biological therapy. Despite therapeutic advances, the 5-year survival rate of gastric cancer is generally <20% (3). Thus, it is necessary to identify more effective therapeutic agents for gastric cancer to improve the survival rate.

**Figure 1:**
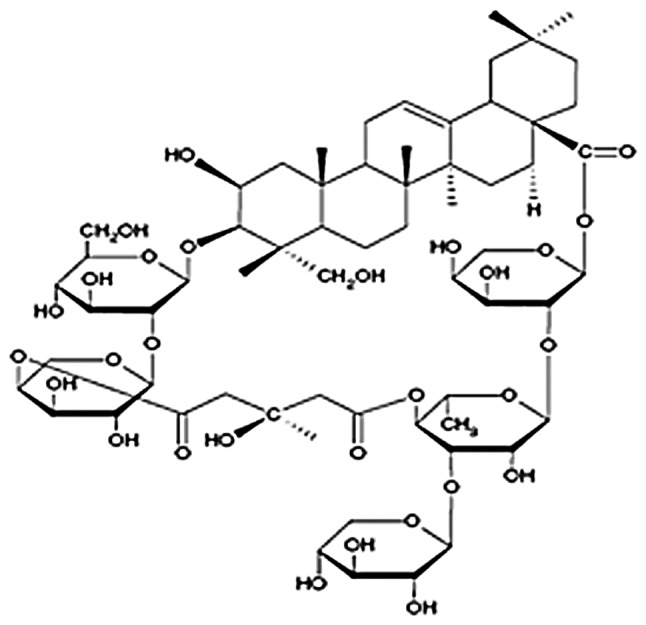
Structure of tubeimoside-1.

A number of studies have drawn attention to natural products extracted from Chinese medicinal herbs as anti-cancer agents in gastric cancer therapy (4–6). Tubeimoside-1 (TBMS1; Fig. 1) is a natural compound isolated from the Chinese medicinal herb *Bolbostemma paniculatum* (Maxim.) Franquet (*Cucurbitaceae*). Previous studies have shown that TBMS1 exhibits a variety of biological activities, including potent anticancer effects in several cancer cell lines, and it has been reported that TBMS1 exerts anticancer effects through the inhibition of cancer cell proliferation and the induction of G2/M phase arrest and apoptosis (7–11). Previously, we reported that TBMS1 inhibits proliferation and induces apoptosis by increasing the Bax to Bcl-2 ratio and decreasing COX-2 expression levels in lung cancer A549 cells (12). However, the effects of TBMS1 on human gastric cancer cells remain unclear.

In the present study, the effects of TBMS1 on the growth of BGC823 gastric cancer cells and the cellular mechanism involved in TBMS1-induced apoptosis were investigated. The findings suggest that TBMS1 may be developed as an anti-cancer agent for gastric cancer therapy.

## Materials and methods

### Reagents and chemicals

TBMS1 was purchased from the National Institute for the Control of Pharmaceutical and Biological Products (Beijing, China) and a 1 mmol/l stock solution of TBMS1 was dissolved in PBS and stored at −20°C. Fetal bovine serum (FBS) was purchased from Solarbio Science and Technology Co., Ltd. (Beijing, China). 3-(4,5-Dimethylthiazol-2-yl)-2,5-diphenyltetrazolium bromide (MTT), Hoechst 33342 and dimethyl sulfoxide (DMSO) were purchased from Sigma-Aldrich (St. Louis, MO, USA). The Annexin V-FITC and PI double staining kit were purchased from KeyGene (Nanjing, China). Antibodies were purchased from Santa Cruz Biotechnology Inc. (Santa Cruz, CA, USA). All other reagents were procured locally.

### Cell culture

The human gastric cancer cell line BGC823 was obtained from the China Center for Type Culture Collection (Wuhan, China) and maintained in RPMI-1640 supplemented with 10% FBS, 100 U/ml penicillin and 100 *μ*g/ml streptomycin at 37°C in a humidified atmosphere of 5% CO_2_.

### MTT assay

The effect of TBMS1 on the proliferation of BGC823 cells was measured by MTT assay. Briefly, BGC823 cells were plated at a density of 1×10^4^ cells per well in 96-well plates overnight and then treated with various concentrations of TBMS1 (0, 5, 10, 15 and 20 *μ*mol/l) for 24 and 48 h. MTT solution (20 *μ*l, 2 mg/ml in PBS) was added to each well and the cells were cultured for a further 4 h at 37°C. The medium was then removed and 150 *μ*l DMSO was added to solubilize the MTT formazan crystals. Finally, the plates were agitated and the optical density was determined at 570 nm (OD570) using an ELISA plate reader (Model 550, Bio-Rad, Hercules, CA, USA). At least three independent experiments were performed.

### Fluorescence microscopy

BGC823 cells (1×10^6^) were seeded in 6-well plates overnight and then treated with different concentrations of TBMS1 (0 and 10 *μ*mol/l) for 24 h. The cells were washed twice with cold PBS, fixed with cold methanol and acetic acid (3/1, v/v) for 30 min and then stained with Hoechst 33342 (1 mg/ml) for 30 min in the dark. The stained cells were observed with a fluorescence microscope (×400 magnification, Nikon, Tokyo, Japan).

### Flow cytometric analysis

The apoptotic rates of the BGC823 cells were determined by flow cytometric analysis using an Annexin V-FITC Apoptosis kit. Briefly, BGC823 cells (1×10^6^) were seeded in 6-well plates overnight and then treated with various concentrations of TBMS1 (0, 5, 10 and 15 *μ*mol/l) for 24 h. Cells (1×10^6^) were then harvested by centrifuging (1,000 rpm) and washed twice with cold PBS. The staining was performed according to the instructions of the manufacturer (KeyGene) and then the cells were analyzed using a FACScan flow cytometer (Becton-Dickinson, San Jose, CA, USA). At least three independent experiments were performed.

### Western blot analysis

The expression of apoptosis-related proteins was evaluated by western blot analysis. Briefly, BGC823 cells (1×10^6^) were seeded in 6-well plates overnight, then treated with various concentrations of TBMS1 (0, 5, 10 and 15 *μ*mol/l). After treatment for 24 h, the total proteins were solubilized and extracted with lysis buffer (20 mM HEPES, pH 7.9, 20% glycerol, 200 mM KCl, 0.5 mM EDTA, 0.5% NP-40, 0.5 mM DTT and 1% protease inhibitor cocktail). The protein concentration was determined using a bicinchoninic acid (BCA) protein assay. All samples were separated by SDS-PAGE to determine the expression levels of Bax, Bcl-2 and β-actin proteins. Blots were developed using an ECL kit.

### Statistical analysis

Statistical analyses were performed using the SPSS 13.0 package (SPSS Inc., Chicago, IL, USA). All experiments were conducted at least three times. All data are expressed as the mean ± SD. The statistical correlations of the data were tested for significance using ANOVA and the Student’s t-test. P<0.05 and P<0.01 were considered to indicate statistically significant differences.

## Results

### TBMS1 inhibited BGC823 cell proliferation

**Figure 2:**
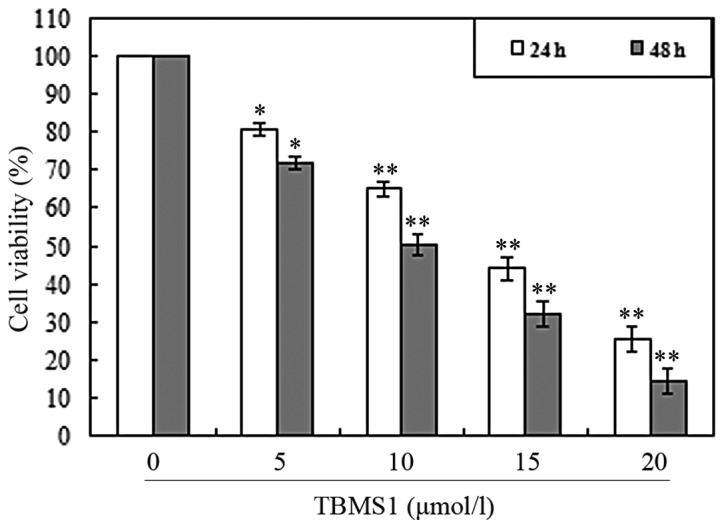
The proliferation inhibiting effects of TBMS1 on BGC823 gastric cancer cells. ^*^P<0.05 vs. the control group. ^**^P<0.01 vs. the control group. TBMS1, tubeimoside-1.

To investigate the growth inhibiting effects of TBMS1, the BGC823 cells were treated with various concentrations of TBMS1 for 24 and 48 h and the rate of inhibition was determined by MTT assays. As shown in Fig. 2, it was observed that the growth of BGC823 cells was inhibited in a concentration- and time-dependent manner.

### TBMS1 induced BGC823 cell apoptosis

**Figure 3:**
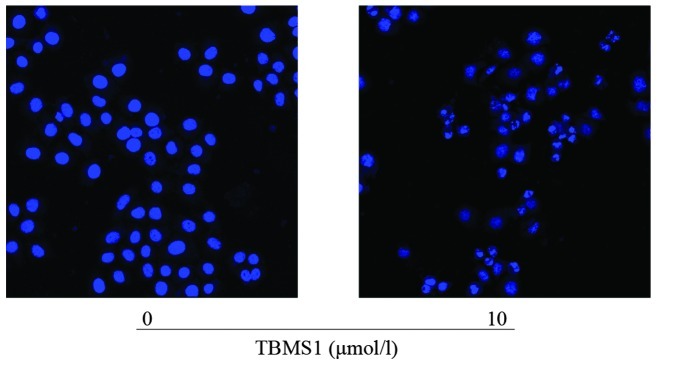
Cell apoptosis observed by Hoechst 33342 staining. BGC823 cells were treated with TBMS1 (0 or 10 *μ*mol/l) for 24 h. Apoptotic cells exhibited chromatin condensation, nuclear fragmentation and apoptotic bodies. TBMS1, tubeimoside-1.

To investigate the apoptosis-inducing effect of TBMS1, the BGC823 cells were treated with various concentrations of TBMS1. After treatment with TBMS1 (0 and 10 *μ*mol/l) for 24 h, the cells were examined by fluorescent microscopy using Hoechst 33324 staining. As shown in Fig. 3, chromatin condensation, nuclear fragmentation and apoptotic bodies were observed clearly in the treated cells. The results revealed that when exposed to TBMS1, BGC823 cells underwent the typical morphological changes of apoptosis.

**Figure 4:**
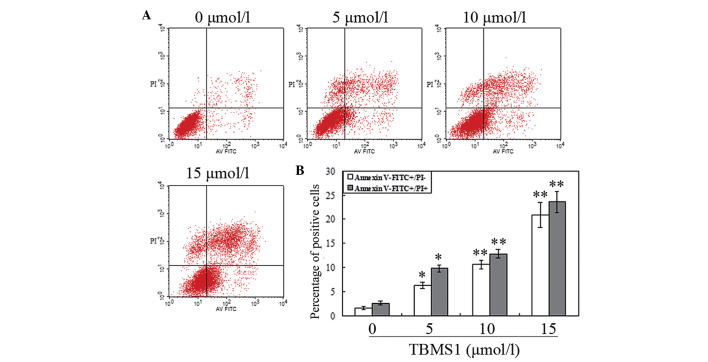
(A) TBMS1-induced apoptosis in BGC823 cells as assayed by flow cytometry. BGC823 cells were treated with TBMS1 (0, 5, 10 and 15 *μ*mol/l) for 24 h. The cells were then harvested and stained with Annexin V and PI and flow cytometric analysis was performed to analyze the apoptosis. (B) Summary of the apoptosis data in histogram form. ^*^P<0.05 vs. the control group; ^**^P<0.01 vs. the control group. TBMS1, tubeimoside-1.

The ratio of apoptotic cells induced by TBMS1 was measured by flow cytometry. BGC823 cells were treated with various concentrations of TBMS1 (0, 5, 10 and 15 *μ*mol/l) for 24 h and analyzed by flow cytometry using Annexin V and PI staining. As shown in Fig. 4, the ratio of early and late apoptotic cells was significantly increased in the TBMS1-treated cells compared with the control group. The results show that when treated with TBMS1 for 24 h, the ratio of apoptotic cells significantly increased in a concentration-dependent manner.

### Effect of TBMS1 on expression levels of the Bcl-2 gene family

**Figure 5:**
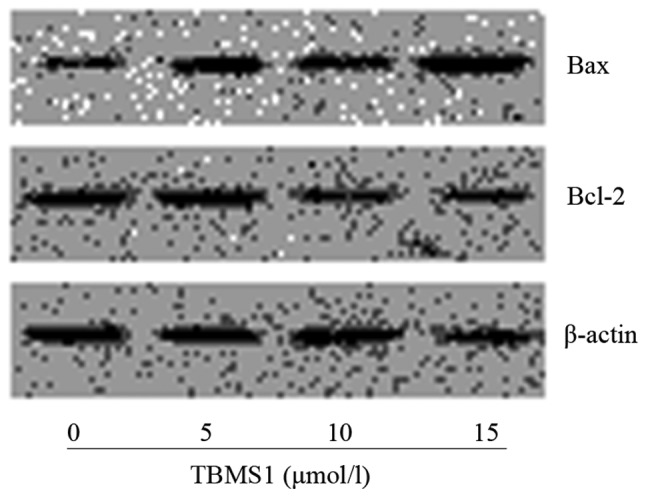
Effect of TBMS1 on Bcl-2 family proteins by western blot analysis. BGC823 cells were treated with TBMS1 (0, 5, 10 and 15 *μ*mol/l) for 24 h. Proteins were extracted, then Bax, Bcl-2 and β-actin expression levels were analyzed by western blotting. TBMS1, tubeimoside-1.

The expression of apoptosis-related proteins was evaluated by western blot analysis. As shown in Fig. 5, TBMS1 treatment led to an increase in Bax levels and a reduction in Bcl-2 levels compared with those in the control cells. The ratio of Bax to Bcl-2 increased in a concentration-dependent manner.

## Discussion

An increasing amount of attention has been focused on the use of natural products isolated from Chinese medicinal herbs for gastric cancer therapy (4–6). TBMS1 is a natural compound extracted from the Chinese medicinal herb *Bolbostemma paniculatum* (Maxim.) Franquet (*Cucurbitaceae*), which has been used for a long time in the treatment of numerous diseases and possesses well-documented antiviral, anti-inflammatory and immunosuppressive activities (13–15). The anticancer effects of TBMS1 have been documented in numerous types of human cancer (7–12). These studies have revealed that TBMS1 inhibits cell growth and induces G2/M phase arrest and the apoptosis of cancer cells. However, the effects of TBMS1 on human gastric cancer cells remain unclear.

In the present study, TBMS1 inhibited BGC823 gastric cancer cell proliferation in a concentration- and time-dependent manner. Chromosome condensation, nuclear fragmentation and apoptotic bodies were observed by fluorescent microscopy. Flow cytometric analysis revealed that TBMS1 induced BGC823 cell apoptosis in a concentration-dependent manner. Moreover, the results from the western blot analysis showed that the molecular basis of the TBMS1-induced apoptosis in the BGC823 cells was via the downregulation of Bcl-2 protein levels and the upregulation of Bax protein expression.

Apoptosis, or programmed cell death, is critical in developmental processes, the maintenance of homeostasis and elimination of damaged cells (16,17). Resistance to apoptosis is a significant hallmark of cancer cells and the induction of apoptosis is one of the major goals of anticancer therapy (18). Several genes are involved in the regulation of apoptosis, such as the Bcl-2 gene family. The Bcl-2 gene family, which is significantly involved in the regulation of cell apoptosis, includes anti-apoptotic genes such as Bcl-2 and Bcl-xl and pro-apoptotic genes including, Bax, Bak, Bik, Bid and Bad (19,20). The ratio of Bax to Bcl-2 is a decisive factor for the induction of apoptosis and the balance between the expression levels of the proteins Bax and Bcl-2 is critical for cell survival or death. Certain anticancer drugs extracted from Chinese medicinal herbs induce cancer cell apoptosis through the upregulation of the ratio of Bax to Bcl-2 (21–24). Similarly, the present results showed that the expression of Bax was upregulated by TBMS1, whereas that of Bcl-2 was downregulated, leading to an upregulation of the ratio between Bax and Bcl-2. This indicates the involvement of the Bcl-2 gene family in the regulation of TBMS1-induced cell apoptosis.

In conclusion, the present study demonstrated that TBMS1 inhibited proliferation and promoted apoptosis in BGC823 gastric cancer cells. This apoptotic response is associated with the regulation of the expression of the Bcl-2 gene family. The findings indicate that TBMS1 may be developed as a possible therapeutic agent for the management of gastric cancer.
